# Application of a correlation correction factor in a microarray cross-platform reproducibility study

**DOI:** 10.1186/1471-2105-8-447

**Published:** 2007-11-15

**Authors:** Kellie J Archer, Catherine I Dumur, G Scott Taylor, Michael D Chaplin, Anthony Guiseppi-Elie, Geraldine Grant, Andrea Ferreira-Gonzalez, Carleton T Garrett

**Affiliations:** 1 Department of Biostatistics, Virginia Commonwealth University, 730 East Broad St., Richmond, VA, USA; 2 Department of Pathology, Virginia Commonwealth University, Richmond, VA, USA; 3 Center for Bioelectronics, Biosensors and Biochips, School of Engineering, Virginia Commonwealth University, Richmond, VA, USA; 4 Center for the Study of Biological Complexity, Virginia Commonwealth University, Richmond, VA, USA; 5 Molecular and Microbiological Department, George Mason University, Manassas, VA, USA

## Abstract

**Background:**

Recent research examining cross-platform correlation of gene expression intensities has yielded mixed results. In this study, we demonstrate use of a correction factor for estimating cross-platform correlations.

**Results:**

In this paper, three technical replicate microarrays were hybridized to each of three platforms. The three platforms were then analyzed to assess both intra- and cross-platform reproducibility. We present various methods for examining intra-platform reproducibility. We also examine cross-platform reproducibility using Pearson's correlation. Additionally, we previously developed a correction factor for Pearson's correlation which is applicable when *X *and *Y *are measured with error. Herein we demonstrate that correcting for measurement error by estimating the "disattenuated" correlation substantially improves cross-platform correlations.

**Conclusion:**

When estimating cross-platform correlation, it is essential to thoroughly evaluate intra-platform reproducibility as a first step. In addition, since measurement error is present in microarray gene expression data, methods to correct for attenuation are useful in decreasing the bias in cross-platform correlation estimates.

## Background

Previous microarray gene expression studies have examined within-platform reproducibility among different generations of the Affymetrix GeneChip [1,2] and among cDNA-based array platforms [3,4]. Subsequently, several cross-platform reproducibility studies have been reported, many of which examined either the consistency of intensities or the consistency with which different platforms identify genes significantly differently expressed [5-18]. Results from another large cross-platform study, the MicroArray Quality Control (MAQC) project, led by the US Food and Drug Administration with 51 participating universities and major biotechnology companies, have also been reported [19-24]. Some of these early studies demonstrated poor cross-platform correlations. For example, among 384 genes commonly declared present in a cDNA-based microarray and the Affymetrix HG-U95Av2 GeneChip platform, the Spearman correlation was only 0.131. Other cross-platform studies also reported low cross-platform correlations [5,8]. In addition, in a study examining three microarray platforms in ten laboratories, correlations between Affymetrix and two-channel arrays ranged from 0.13 – 0.57 [25]. More recent research has demonstrated that poor correlations may be observed when at least one platform under examination suffers from low intra-platform reproducibility or when a poor data analytic method is applied [26].

Most of these studies estimated Pearson's correlation as a means of assessing cross-platform reproducibility. That is, we consider *X *and *Y *to be microarray gene expression values from two different platforms, and *ρ*_*XY *_is estimated. However, for microarray data, both random variables *X *and *Y *are subject to measurement error. It is well known that the flourescent intensities from the scanned microarray images are proxies for the true underlying gene expression values [27]. Therefore, microarray gene expression values are measured with error. When examining cross-platform correlation, inconsistencies in measured intensities can be due to systematic platform biases as well as random intra-platform variability. Statistical methods that account for measurement error (ME), such as regression calibration, have been applied in a variety of scenarios to correct for the known bias caused by ME in parameter estimation [28]. In a recent review, the authors stated that within the next 5 years, "calibration methods will be introduced to systematically correct ratio underestimation by microarray technology" [29]. We have undertaken such an effort to account for the random intra-platform variability by developing a "disattenuated" correlation estimate [30] which accounts for random intra-platform variation in both *X *and *Y*, and demonstrate its use in measuring cross-platform correlation.

Microarray hybridizations were performed using three different technologies, each in a different laboratory. The Affymetrix (Affy) HG-U133A GeneChip was utilized in the Virginia Commonwealth University's (VCU) Division of Molecular Diagnostics Laboratory. A custom-designed oligonucleotide microarray designed specifically to interrogate genes more commonly expressed in brain tissue was used in VCU's School of Engineering's Center for Bioelectronics, Biosensors and Biochips (C3B). The C3B microarray platform comprises 10,000 genes represented by 3' fifty-mer oligonucleotides (MWG Biotech) that were spotted in duplicate. Finally, a cDNA microarray spotted with full and partial length PCR probes (Research Genetics/Invitrogen) was used in George Mason University's (GMU) Center for Biomedical Genomics and Informatics.

Each laboratory designed a small experiment to assess intra-platform quality control. Each laboratory used the same lot of reference RNA, the Stratagene Total Human RNA, for hybridizing a set of technical replicates for a process variability study. These 'self-self' hybridizations permit meaningful assessments of reproducibility since, under ideal circumstances such as that the same experimental conditions exist among platforms and that there are no probe-binding affinity effects, each gene across the set of chips should exhibit linearly related gene expression intensities across platforms. Although the RNA hybridized was from the same lot, the study designs and protocols differed from lab to lab. A description of of each experiment can be found in the Methods section of this paper.

## Results

### Within-platform comparisons

**Figure 1:**
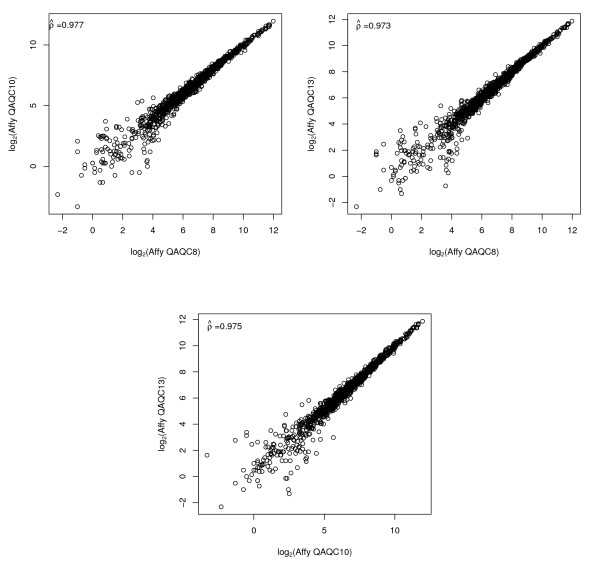
**Affymetrix**. Pairwise scatterplots and Pearson's correlation for Affymetrix GeneChips (MAS5 summaries) restricted to the 1,288 genes in common among the three platforms.

**Figure 2:**
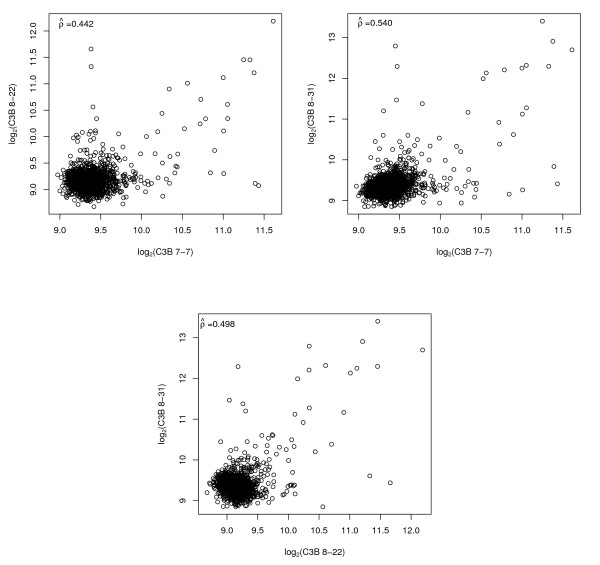
**C3B**. Pairwise scatterplots and Pearson's correlationfor C3B arrays restricted to the 1,288 genes in common among the three platforms.

**Figure 3:**
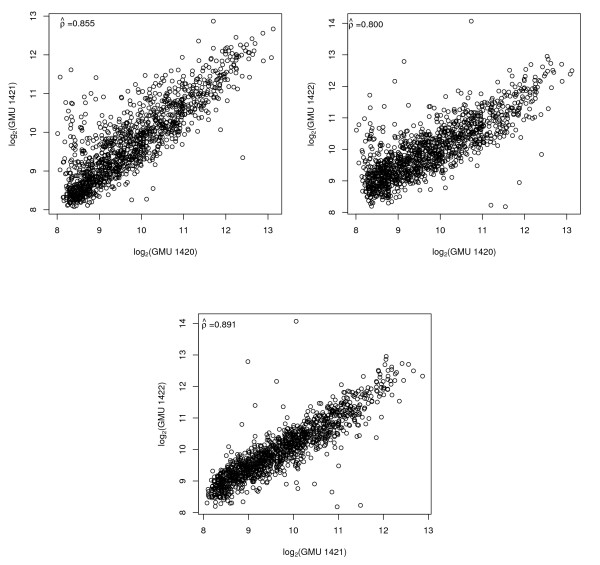
**GMU**. Pairwise scatterplots and Pearson's correlation for GMU arrays restricted to the 1,288 genes in common among the three platforms.

Prior to estimating cross-platform correlations, we performed a thorough examination of intra-platform reproducibility, as recommended [29]. Since the Stratagene Total Human RNA was used as both the experimental and reference sample, the expected log_2 _ratio for all genes is 1, so that no correlation is expected when comparing two arrays in terms of the log_2 _ratio. Therefore for two channel arrays, we restricted attention to intensities from one channel as well as to the post-normalized intensities from that same channel. For the Affymetrix GeneChip, intensities were highly correlated across the set of three technical replicates for all expression summary methods (Table 1 and Figure 1). The GMU arrays were strongly correlated, though the C3B arrays were not highly correlated (Figures 2 and 3).

**Table 1:** Average correlation for the Affymetrix, C3B, and GMU Stratagene Technical Replicates dataset for various expression summary methods.

Platform	Expression Summary Method	Average Correlation
Affymetrix	MAS 5.0	0.9955
(*N *= 22,283)	RMA	0.9994
	GC-RMA	0.9998
C3B	Channel 1 foreground	0.6560
(*N *= 21,168)	Print-tip loess normalized Ch1	0.6593
GMU	Cy5 foreground	0.6236
(*N *= 21,168)	Print-tip loess normalized Cy5	0.8475

The weighted kappa statistics indicated that the Affymetrix platform had the highest agreement among ranked intensities (Table 2), followed by the GMU array which also exhibited good agreement among the technical replicates when considering the ranked gene intensities. The weighted kappa statistics for C3B platform suggested the ranked intensities from the three technical replicates were not in agreement, yielding an insignificant p-value for two of the array comparisons. A similar conclusion, that the Affymetrix platform followed by the GMU array demonstrated the highest reproducibility, with low reproducibility among the C3B arrays, was noted upon examination of the proportion of invariant features (Table 3). Although intra-platform reproducibility varied among the three platforms studied, all platforms yield gene expression intensities that are subject to some degree of measurement error.

**Table 2:** Observed agreement and p-value for each pairwise comparison within each platform using the weighted kappa statistic. Print-tip loess normalized Cy5 intensities were used for both two-channel arrays; MAS5.0 expression summaries were used for Affymetrix GeneChips.

Chips compared	Observed Agreement	P-value
GMU		
1420 v 1421	71.89%	< 0.0001
1420 v 1422	66.31%	< 0.0001
1421 v 1422	75.41%	< 0.0001
C3B		
7–7 v 8–22	28.18%	1.00
7–7 v 8–31	51.82%	< 0.0001
8–22 v 8–31	29.14%	1.00
Affymetrix		
QAQC8 v QAQC10	89.75%	< 0.0001
QAQC8 v QAQC13	89.54%	< 0.0001
QAQC10 v QAQC13	89.57%	< 0.0001

**Table 3:** Frequency and percent of invariant features from each platform (*P *< 0.0001). Print-tip loess normalized Cy5 intensities were used for both two-channel arrays; MAS5.0 expression summaries were used for Affymetrix GeneChips.

	Invariant	Not Invariant
	N, (%)	N, (%)
Affymetrix	11,732 (52.65%)	10,551 (47.35%)
C3B	594 (2.81%)	20,574 (97.19%)
GMU	3,036 (14.34%)	18,132 (85.66%)

### Cross-platform comparisons

For the GMU array the 21,168 spots correspond to 19,894 distinct clones, with the feature name of each spot denoted by Unigene ID. There were 2,744 Affy probe sets that matched a GMU Unigene ID. Among these, 145 Unigene IDs were interrogated by more than one probe set. After restricting attention to unique clones and probes sets there were 2,587 unique probe sets/clones in common to GMU and the Affy platforms. For the C3B arrays, since its design is essentially two identical subarrays laid out in duplicate with the feature name of each spot denoted by RefSeqID, the average expression for each RefSeqID was calculated prior to merging the spots with the Affymetrix probe sets. That is, the 21,168 long oligos correspond to 10,040 distinct genes. For the C3B array, there were 9,000 distinct RefSeqIDs were interrogated by at least one Affymetrix probe set meeting our criteria. Once the data from the two different 2-channel arrays were merged to the Affymetrix GeneChip data (i.e., GMU-Affy and C3B-Affy), these two resulting datasets were then merged by Affymetrix probe set ID, resulting in 1,288 common probe sets/spots among the three platforms.

Not accounting for measurement error, the average Pearson correlations (ρ¯
 MathType@MTEF@5@5@+=feaafiart1ev1aaatCvAUfKttLearuWrP9MDH5MBPbIqV92AaeXatLxBI9gBaebbnrfifHhDYfgasaacPC6xNi=xH8viVGI8Gi=hEeeu0xXdbba9frFj0xb9qqpG0dXdb9aspeI8k8fiI+fsY=rqGqVepae9pg0db9vqaiVgFr0xfr=xfr=xc9adbaqaaeGacaGaaiaabeqaaeqabiWaaaGcbaacciGaf8xWdiNbaebaaaa@2DB2@_*w*_) of the log transformed Affymetrix GeneChip expression and C3B array expression are reported in Table 4 for MAS 5.0, RMA, and GC-RMA expression summaries as 'naïve' estimates of correlation. In addition, the disattenuated correlations (ρ˜
 MathType@MTEF@5@5@+=feaafiart1ev1aaatCvAUfKttLearuWrP9MDH5MBPbIqV92AaeXatLxBI9gBaebbnrfifHhDYfgasaacPC6xNi=xH8viVGI8Gi=hEeeu0xXdbba9frFj0xb9qqpG0dXdb9aspeI8k8fiI+fsY=rqGqVepae9pg0db9vqaiVgFr0xfr=xfr=xc9adbaqaaeGacaGaaiaabeqaaeqabiWaaaGcbaacciGaf8xWdiNbaGaaaaa@2DA9@), obtained when considering that the C3B and Affy gene intensities are subject to measurement error, are also reported. Noting that the attenuation for the C3B arrays is 0.386, that is, over half of the variability is attributed to measurement error, the disattentuated correlations estimated using measurement error models are substantially higher, irrespective of the Affymetrix expression summary method used. This suggests that previous use of Pearson's correlation under-estimated true underlying cross-platform correlations. That is, the effect of the presence of random intra-platform variation is degraded performance on the apparent cross-platform correlation. Therefore, by removing random intra-platform variation through measurement error methodology, the cross-platform correlation will go up.

**Table 4:** Cross-platform average Pearson correlations (ρ¯
 MathType@MTEF@5@5@+=feaafiart1ev1aaatCvAUfKttLearuWrP9MDH5MBPbIqV92AaeXatLxBI9gBaebbnrfifHhDYfgasaacPC6xNi=xH8viVGI8Gi=hEeeu0xXdbba9frFj0xb9qqpG0dXdb9aspeI8k8fiI+fsY=rqGqVepae9pg0db9vqaiVgFr0xfr=xfr=xc9adbaqaaeGacaGaaiaabeqaaeqabiWaaaGcbaacciGaf8xWdiNbaebaaaa@2DB2@_*w*_) and disattenuated cross-platform correlations (ρ˜
 MathType@MTEF@5@5@+=feaafiart1ev1aaatCvAUfKttLearuWrP9MDH5MBPbIqV92AaeXatLxBI9gBaebbnrfifHhDYfgasaacPC6xNi=xH8viVGI8Gi=hEeeu0xXdbba9frFj0xb9qqpG0dXdb9aspeI8k8fiI+fsY=rqGqVepae9pg0db9vqaiVgFr0xfr=xfr=xc9adbaqaaeGacaGaaiaabeqaaeqabiWaaaGcbaacciGaf8xWdiNbaGaaaaa@2DA9@) for Stratagene Technical Replicate Dataset using MAS 5.0, RMA, and GC-RMA Affy expression summaries.

	MAS 5.0		RMA		GC-RMA	
Platform	ρ¯ MathType@MTEF@5@5@+=feaafiart1ev1aaatCvAUfKttLearuWrP9MDH5MBPbIqV92AaeXatLxBI9gBaebbnrfifHhDYfgasaacPC6xNi=xH8viVGI8Gi=hEeeu0xXdbba9frFj0xb9qqpG0dXdb9aspeI8k8fiI+fsY=rqGqVepae9pg0db9vqaiVgFr0xfr=xfr=xc9adbaqaaeGacaGaaiaabeqaaeqabiWaaaGcbaacciGaf8xWdiNbaebaaaa@2DB2@_*w*_	ρ˜ MathType@MTEF@5@5@+=feaafiart1ev1aaatCvAUfKttLearuWrP9MDH5MBPbIqV92AaeXatLxBI9gBaebbnrfifHhDYfgasaacPC6xNi=xH8viVGI8Gi=hEeeu0xXdbba9frFj0xb9qqpG0dXdb9aspeI8k8fiI+fsY=rqGqVepae9pg0db9vqaiVgFr0xfr=xfr=xc9adbaqaaeGacaGaaiaabeqaaeqabiWaaaGcbaacciGaf8xWdiNbaGaaaaa@2DA9@	ρ¯ MathType@MTEF@5@5@+=feaafiart1ev1aaatCvAUfKttLearuWrP9MDH5MBPbIqV92AaeXatLxBI9gBaebbnrfifHhDYfgasaacPC6xNi=xH8viVGI8Gi=hEeeu0xXdbba9frFj0xb9qqpG0dXdb9aspeI8k8fiI+fsY=rqGqVepae9pg0db9vqaiVgFr0xfr=xfr=xc9adbaqaaeGacaGaaiaabeqaaeqabiWaaaGcbaacciGaf8xWdiNbaebaaaa@2DB2@_*w*_	ρ˜ MathType@MTEF@5@5@+=feaafiart1ev1aaatCvAUfKttLearuWrP9MDH5MBPbIqV92AaeXatLxBI9gBaebbnrfifHhDYfgasaacPC6xNi=xH8viVGI8Gi=hEeeu0xXdbba9frFj0xb9qqpG0dXdb9aspeI8k8fiI+fsY=rqGqVepae9pg0db9vqaiVgFr0xfr=xfr=xc9adbaqaaeGacaGaaiaabeqaaeqabiWaaaGcbaacciGaf8xWdiNbaGaaaaa@2DA9@	ρ¯ MathType@MTEF@5@5@+=feaafiart1ev1aaatCvAUfKttLearuWrP9MDH5MBPbIqV92AaeXatLxBI9gBaebbnrfifHhDYfgasaacPC6xNi=xH8viVGI8Gi=hEeeu0xXdbba9frFj0xb9qqpG0dXdb9aspeI8k8fiI+fsY=rqGqVepae9pg0db9vqaiVgFr0xfr=xfr=xc9adbaqaaeGacaGaaiaabeqaaeqabiWaaaGcbaacciGaf8xWdiNbaebaaaa@2DB2@_*w*_	ρ˜ MathType@MTEF@5@5@+=feaafiart1ev1aaatCvAUfKttLearuWrP9MDH5MBPbIqV92AaeXatLxBI9gBaebbnrfifHhDYfgasaacPC6xNi=xH8viVGI8Gi=hEeeu0xXdbba9frFj0xb9qqpG0dXdb9aspeI8k8fiI+fsY=rqGqVepae9pg0db9vqaiVgFr0xfr=xfr=xc9adbaqaaeGacaGaaiaabeqaaeqabiWaaaGcbaacciGaf8xWdiNbaGaaaaa@2DA9@
C3B	0.240	0.391	0.210	0.338	0.168	0.270
GMU	0.384	0.428	0.348	0.383	0.399	0.440

The average Pearson correlations (ρ¯
 MathType@MTEF@5@5@+=feaafiart1ev1aaatCvAUfKttLearuWrP9MDH5MBPbIqV92AaeXatLxBI9gBaebbnrfifHhDYfgasaacPC6xNi=xH8viVGI8Gi=hEeeu0xXdbba9frFj0xb9qqpG0dXdb9aspeI8k8fiI+fsY=rqGqVepae9pg0db9vqaiVgFr0xfr=xfr=xc9adbaqaaeGacaGaaiaabeqaaeqabiWaaaGcbaacciGaf8xWdiNbaebaaaa@2DB2@_*w*_) of the log transformed Affymetrix GeneChip expression and GMU array expression are also reported in Table 4 for MAS 5.0, RMA, and GC-RMA expression summaries, as well as the disattenuated correlations (ρ˜
 MathType@MTEF@5@5@+=feaafiart1ev1aaatCvAUfKttLearuWrP9MDH5MBPbIqV92AaeXatLxBI9gBaebbnrfifHhDYfgasaacPC6xNi=xH8viVGI8Gi=hEeeu0xXdbba9frFj0xb9qqpG0dXdb9aspeI8k8fiI+fsY=rqGqVepae9pg0db9vqaiVgFr0xfr=xfr=xc9adbaqaaeGacaGaaiaabeqaaeqabiWaaaGcbaacciGaf8xWdiNbaGaaaaa@2DA9@). The attenuation for the GMU arrays is 0.824, therefore the disattenuated correlations estimated using measurement error models are larger than their corresponding naïve estimates, though not as markedly in comparison to the C3B arrays. This is due to the higher reliability among the GMU expression intensities.

## Discussion

In this paper, both intra- and cross-platform reproducibility was examined for the Affymetrix and two dual channel microarrays (C3B and GMU). We applied various methods for examining within-platform reproducibility including Pearson's correlation, the weighted kappa, and percent of invariant genes. We also examine cross-platform reproducibility using Pearson's correlation. We previously demonstrated the effectiveness of applying a correlation correction factor via a small simulation study and demonstrated its application in estimating gene-specific correlations. In this paper we demonstrated its use in estimating cross-platform reproducibility. We note that correcting for measurement error by estimating the "disattenuated" correlation removes the bias or attenuation inherent in cross-platform correlation estimates. Specifically, to the extent that random intra-platform variation is present, the effect is degraded performance on the apparent cross-platform correlation. Therefore, by removing random intra-platform variation through measurement error methodology, the cross-platform correlation will go up.

Due to the increased public availability of gene expression microarray data through Gene Expression Omnibus [31] and ArrayExpress [32], researchers are increasingly interested in methods that integrate the results from various microarray studies performed on similar types of samples [33-37]. A careful understanding of variability due to platform-specific bias and random intra-platform variability will help investigators select methods for integrating cross-platform results. Specifically, the amount of attenuation for a specific platform could be used as a platform-specific quality measure and incorporated into a meta-analytic framework [38]. Moreover, gene-specific attenuation factors could be used to adjust for quality in a gene-wise fashion in such models.

A major application of DNA microarray technology is differential gene expression profiling, or the detection of the differences in expression levels of genes between two different types of samples. Some have argued that the consistency of the differences via fold-change or ratio is a more relevant metric for assessing cross-platform comparability than intensities from a single channel. However, to estimate the correlation between fold-changes from two platforms, two different samples are needed. We therefore plan to use data from the MAQC project to examine cross-platform fold-change correlations. In addition, it has been suggested that a more relevant metric is not agreement in the identification of individual differentially expressed genes, but rather whether consistent and accurate predictions of sample class is obtained from the platforms being compared [39]. This metric should be included is such cross-platform studies as well.

Previous researchers demonstrated that single and two channel microarrays yield consistent results, and concluded that the selection of which technology to use is not necessarily a critical factor in the design of a microarray study [20]. Here we demonstrate the critical need to thoroughly evaluate intra-platform reproducibility, a finding which has been been noted by others [26]. In this study, we examined two dual channel platforms and the Affymetrix platform. While the C3B and GMU platforms are not widely used by the microarray research community, they do represent a class of microarrays that are commonly used, two channel custom spotted/home brewed arrays. Thus, we believe these results are of general interest to those who use both commercial and custom designed arrays. While the C3B two channel platform had poor reproducibility, the GMU two channel and Affymetrix platforms had good reproducibility. We repeated the intra-platform analysis using the following three sets of randomly selected Affymetrix GeneChips (6, 12, 2), (5, 16, 14), and (5, 2, 3) and the intra-platform Affymetrix results were consistently reproducible with what is presented in this paper. This high reproducibility of the Affymetrix GeneChip data has also been reported by other investigators [14,40]. These data have proven useful in selecting a platform for studying biological specimens being collected by our tissue bank. We recommend that prior to performing expensive microarray hybridizations using irreplacable biological specimens procured from clinical studies, a thorough assessment of intra-platform reproducibility be conducted.

One limitation of this study is that platform is completely confounded with laboratory technician and protocol, that is, the platform-specific sequence of reactions, scanner, procedures and events involved in the production of microarray data. It was previously noted that there is a high positive correlation between technician experience and intra-platform correlation [25]. This is consistent with our findings, whereby a first year graduate student performed the C3B hybridizations (ρ¯
 MathType@MTEF@5@5@+=feaafiart1ev1aaatCvAUfKttLearuWrP9MDH5MBPbIqV92AaeXatLxBI9gBaebbnrfifHhDYfgasaacPC6xNi=xH8viVGI8Gi=hEeeu0xXdbba9frFj0xb9qqpG0dXdb9aspeI8k8fiI+fsY=rqGqVepae9pg0db9vqaiVgFr0xfr=xfr=xc9adbaqaaeGacaGaaiaabeqaaeqabiWaaaGcbaacciGaf8xWdiNbaebaaaa@2DB2@ = 0.656), while the GMU and Affy hybridizations were performed by Ph.D. faculty members (ρ¯
 MathType@MTEF@5@5@+=feaafiart1ev1aaatCvAUfKttLearuWrP9MDH5MBPbIqV92AaeXatLxBI9gBaebbnrfifHhDYfgasaacPC6xNi=xH8viVGI8Gi=hEeeu0xXdbba9frFj0xb9qqpG0dXdb9aspeI8k8fiI+fsY=rqGqVepae9pg0db9vqaiVgFr0xfr=xfr=xc9adbaqaaeGacaGaaiaabeqaaeqabiWaaaGcbaacciGaf8xWdiNbaebaaaa@2DB2@ = 0.848 and ρ¯
 MathType@MTEF@5@5@+=feaafiart1ev1aaatCvAUfKttLearuWrP9MDH5MBPbIqV92AaeXatLxBI9gBaebbnrfifHhDYfgasaacPC6xNi=xH8viVGI8Gi=hEeeu0xXdbba9frFj0xb9qqpG0dXdb9aspeI8k8fiI+fsY=rqGqVepae9pg0db9vqaiVgFr0xfr=xfr=xc9adbaqaaeGacaGaaiaabeqaaeqabiWaaaGcbaacciGaf8xWdiNbaebaaaa@2DB2@ = 0.996, respectively). Future studies that control for external factors that may influence intra-platform reliability are warranted.

**Figure 4:**
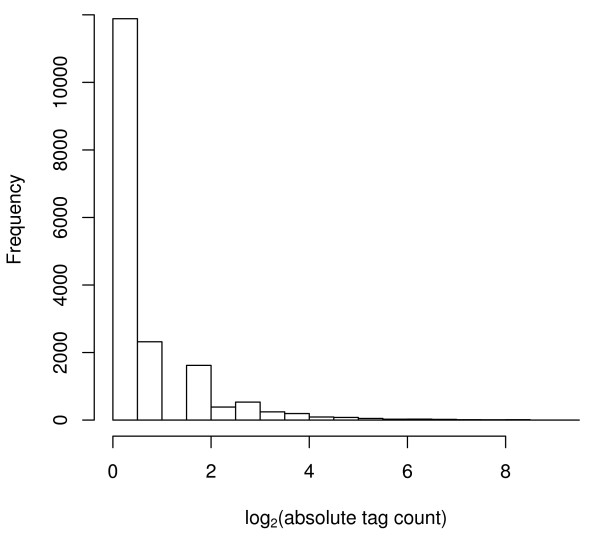
**Histogram of log_2 _absolute tag counts from SAGE**. Histogram of log_2 _absolute tag counts from Serial Analysis of Gene Expression using the Stratagene Total Human RNA for the 14000 unique tags. Data available from GEO Accession #GSM1734.

**Figure 5:**
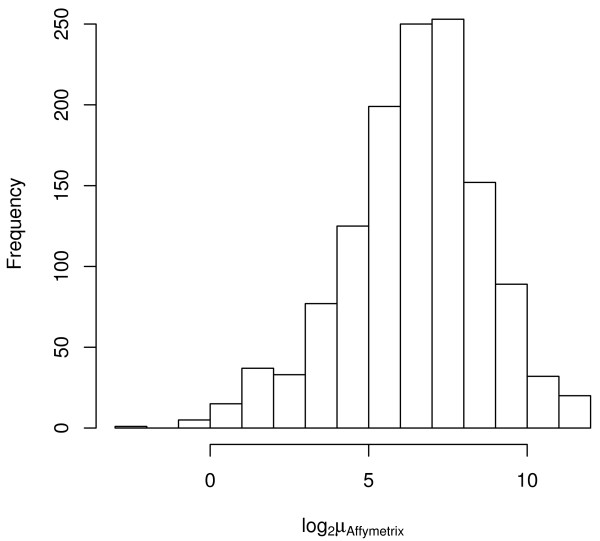
**Histogram of log_2 _average Affymetrix MAS5 signal**. Histogram of log_2 _average Affymetrix MAS5 signal for the Stratagene Total Human RNA using the 1,288 genes in common among the three platforms.

**Figure 6:**
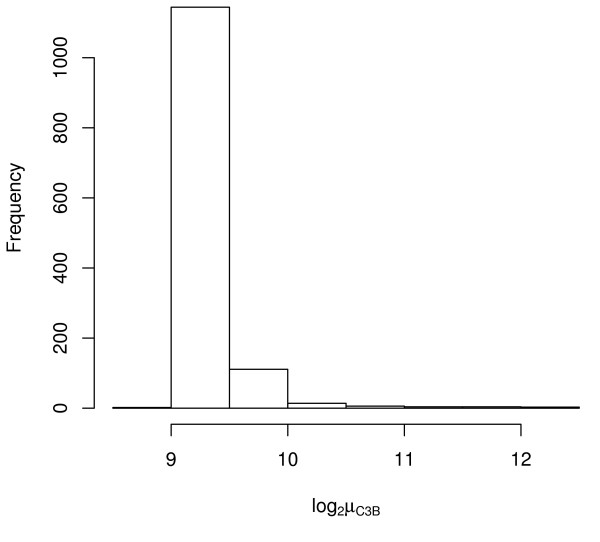
**Histogram of log_2 _average C3B signal**. Histogram of log_2 _average C3B signal for the Stratagene Total Human RNA using the 1,288 genes in common among the three platforms.

**Figure 7:**
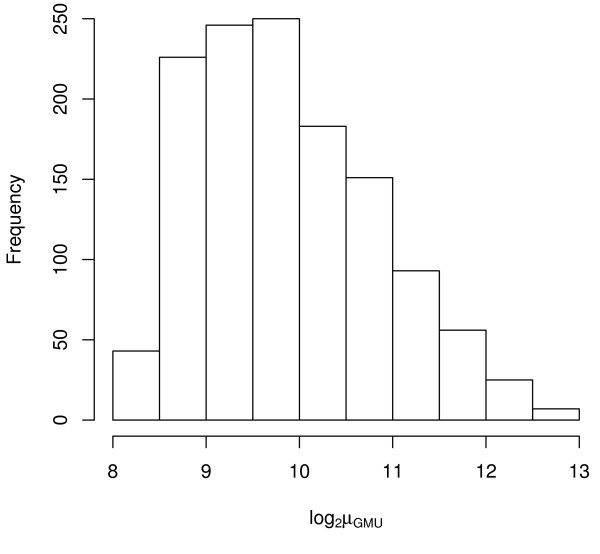
**Histogram of log_2 _average GMU signal**. Histogram of log_2 _average GMU signal for the Stratagene Total Human RNA using the 1,288 genes in common among the three platforms.

In calculating cross-platform correlation, we assumed that the correlation estimated using the using the 1288 matching probes across the three platforms are representative of expected correlation of genes in the human genome that could be represented on the plaforms. Examination of absolute tag counts for the Stratagene Total Human RNA obtained using Serial Analysis of Gene Expression data (available from GEO #GSM1734) revealed that the intensity distribution of the 1,288 genes in common among the three platforms is not representative of the range of expected values (Figures 4, 5, 6, 7). Thus the commonly invoked procedure of estimating cross-platform consistency using only probes in common to all platforms is demonstrated to suffer from bias related to genomic coverage and probe annotation. Future studies comparing commercially available and custom designed arrays need to take this into consideration.

## Conclusion

When estimating cross-platform correlation, it is essential to thoroughly evaluate intra-platform reproducibility as a first step. We also note that the commonly invoked procedure of estimating cross-platform consistency using only probes in common to all platforms is demonstrated to suffer from bias related to genomic coverage and probe annotation. Future studies comparing commercially available and custom designed arrays need to take this into consideration. Moreover, to the extent that random intra-platform variation is present, the effect is degraded performace on the apparent cross-platform correlation. Therefore, by removing random intra-platform variation through measurement error methodology, the cross-platform correlation will go up. Methods to correct for attenuation, such as that presented, are thus useful in decreasing such a bias in cross-platform correlation estimates. Platform-specific attenuation estimates may subsequently be used as a platform-specific quality measure and incorporated into a meta-analytic framework.

## Methods

### Stratagene Technical Replicates Dataset

Previously, each laboratory designed a small experiment to assess intra-platform quality control. Each laboratory used the same lot of reference RNA, the Stratagene Total Human RNA, for hybridizing a set of technical replicates for a process variability study. These 'self-self' hybridizations permit meaningful assessments of reproducibility since, under ideal circumstances such as that the same experimental conditions exist among platforms and that there are no probe-binding affinity effects, each gene across the set of chips should exhibit linearly related gene expression intensities across platforms. Although the RNA hybridized was from the same lot, the study designs and protocols differed from lab to lab.

The Affy platform was assessed using an unbalanced three-factor design using 16 technical replicates [41]. The same reference RNA sample was examined in 16 different chips run on two days in four different modules of the Affymetrix fluidics workstation. Fresh fragmented cRNAs were hybridized to the first four GeneChips on Day 1 while frozen fragmented cRNAs were hybridized to remaining four GeneChips on Day 1 and to all eight GeneChips processed on Day 2. To eliminate operator variations, the same person completed the synthesis and hybridization of all 16 chips. The images were scanned at a 6 *μm *resolution using the Agilent G2500A Technologies Gene Array scanner. The full set of 16 Affymetrix GeneChips is publicly available [42].

At GMU, the RNA was amplified using the MessageAmp aRNA Kit (Ambion). The amplified RNA (aRNA) was quantified and its quality was monitored by agarose gel and average size by the Agilent 2100 Bioanalyzer. The same amount of aRNA (4 *μg*) were labeled with Cy3 and Cy5 according the The Institute for Genomic Research protocol and hybridized to three Human I chips. For each chip, the Stratagene Total Human RNA served as both the experimental and reference sample [43]. The ScanArray Express HT confocal laser scanner with settings at 75% of photomultiplier tube, 75% of laser power, and 10 *μm *of pixel resolution was used. Images were aquired by ScanArray Express 2.0 software and processed with QuantArray software.

The C3B laboratory assessed quality of their fabricated microarray using a fractional factorial design. The factors investigated were cDNA labeling strategy (3 levels: Dye conjugated nucleotide, aminoallyl, and Genesphere dendimer labeling), input total RNA concentration ratio (3 levels: 1:1, 1:2, 1:4), hybridization time (2 levels: 4 and 16 hours), hybridization buffer (3 levels: Genesphere, MWG, and Amersham buffer), and production lot (2 levels: lot 7 and 9). Due to the expense of microarray production and hybridization, a fractional factorial design, rather than the full factorial design, was used. Therefore, all combinations of experimental conditions were not included. Specifically, by assuming that high-order interactions are negligible, information regarding the main effects and low-order interactions may be obtained by running only a fraction of the complete factorial design. Since we were interested in examining the effects of hybridization buffer (3 levels), RNA input ratio (3 levels), labeling strategy (3 levels), hybridization time (2 levels), and lot (2 levels), we were initially interested in a 3^3 ^*× *2^2 ^design. However, due to the expense involved in running a full factorial microarray experiment, a 2^8-2 ^fractional factorial design was adopted with defining relation is I = ABCDG = ABEFH = CDEFGH. This resolution V design permits estimation of all main effects and two-factor interactions under the assumption that three-way and higher order interaction terms may be ignored. Thus our experiment required 64 C3B arrays to be hybridized given the factors and levels of interest. Again, for each array the Stratagene Total Human RNA served as both the experimental and reference sample. Hybridized arrays were scanned with ScanArray Express microarray scanner (Perkin Elmer) at 80% laser power, 70% PMT gain, and 5 *μm *scan resolution. Spot intensities were acquired from the images using QuantArray software.

The analyses conducted in the current study were restricted to an equal number of chips by platform to ensure one technology did not dominate the results simply because of having a larger sample size. Three arrays were hybridized at GMU, so a random sample of size 3 was taken from the 16 Affy hybridized samples. These three GeneChips were QAQC8.CEL (Day 1 Frozen), QAQC10.CEL (Day 2 Frozen), and QAQC13.CEL (Day 2 Frozen). The three replicates selected from the C3B fractional factorial study were chosen based on 'optimal' hybridization conditions identified from the fractional factorial experiment. Specifically, the number of genes found to be signficantly different from the analysis of variance model was used as the metric estimating the relative influence of each main and two-factor interaction term. The level of each factor having the smallest number of genes differentially expressed was considered optimal. The three C3B chips used in this study were hybridized using the same buffer (Amersham), ratio of input experimental and control samples (1:1), and labeling method (Aminoallyl Post RT). The chips differed with respect to lot number and hybridization time, though these factors were found to not significantly influence the resulting intensities in the larger study.

### Normalization

Since single-channel arrays measure expression intensities on an absolute scale whereas two-channel arrays measure expression intensities on a ratio-metric scale, we first investigated intra-platform reproducibility using different methods for calculating gene expression to aid in our determination of how to best transform the intensities from the three platforms to a similar scale. In addition, since the objective included an assessment of platform-specific reproducibility across the set of available technical replicates, methods for within-array normalization rather than methods that simultaneously normalize the data across all arrays, were applied in a platform-specific fashion.

For the two-channel arrays, we employed a commonly used procedure of normalizing the spot-level intensities on the array using print-tip loess regression and the subsequently analyzing the normalized spot-level intensities [44]. The use of normalized spot intensities has removed the systematic sources of variability (or at least, reduced) attributed to technical artifacts of no interest, such as deposition differences, differences in labeling efficiencies, print-tip differences etc. Specifically, due to spot differences attributed to deposition gain, print-tip, and dye effects noted among two-channel arrays, each two-channel array (C3B and GMU) was normalized by estimating the corrections M^i
 MathType@MTEF@5@5@+=feaafiart1ev1aaatCvAUfKttLearuWrP9MDH5MBPbIqV92AaeXatLxBI9gBaebbnrfifHhDYfgasaacPC6xNi=xH8viVGI8Gi=hEeeu0xXdbba9frFj0xb9qqpG0dXdb9aspeI8k8fiI+fsY=rqGqVepae9pg0db9vqaiVgFr0xfr=xfr=xc9adbaqaaeGacaGaaiaabeqaaeqabiWaaaGcbaGafmyta0KbaKaadaWgaaWcbaGaemyAaKgabeaaaaa@2E8D@ for spots *i *= 1, ..., *G *by fitting print-tip loess regression models to the *M*_*i *_= *log*_2_(channel 1_*i*_/channel 2_*i*_) (log difference) on *A*_*i *_= (*log*_2_(channel 1_*i*_) + *log*_2_(channel 2_*i*_))/2 (log average) [45]. Probe intensities were then adjusted by Minorm=Mi−M^i
 MathType@MTEF@5@5@+=feaafiart1ev1aaatCvAUfKttLearuWrP9MDH5MBPbIqV92AaeXatLxBI9gBaebbnrfifHhDYfgasaacPC6xNi=xH8viVGI8Gi=hEeeu0xXdbba9frFj0xb9qqpG0dXdb9aspeI8k8fiI+fsY=rqGqVepae9pg0db9vqaiVgFr0xfr=xfr=xc9adbaqaaeGacaGaaiaabeqaaeqabiWaaaGcbaGaemyta00aa0baaSqaaiabdMgaPbqaaiabd6gaUjabd+gaVjabdkhaYjabd2gaTbaakiabg2da9iabd2eannaaBaaaleaacqWGPbqAaeqaaOGaeyOeI0Iafmyta0KbaKaadaWgaaWcbaGaemyAaKgabeaaaaa@3B85@, therefore, Minorm
 MathType@MTEF@5@5@+=feaafiart1ev1aaatCvAUfKttLearuWrP9MDH5MBPbIqV92AaeXatLxBI9gBaebbnrfifHhDYfgasaacPC6xNi=xH8viVGI8Gi=hEeeu0xXdbba9frFj0xb9qqpG0dXdb9aspeI8k8fiI+fsY=rqGqVepae9pg0db9vqaiVgFr0xfr=xfr=xc9adbaqaaeGacaGaaiaabeqaaeqabiWaaaGcbaGaemyta00aa0baaSqaaiabdMgaPbqaaiabd6gaUjabd+gaVjabdkhaYjabd2gaTbaaaaa@341A@ represents the normalized log ratios [46]. In addition, to enforce an absolute expression measure, the normalized ratios were subsequently transformed to yield the channel 1 normalized intensities by xinorm=2Ai+Minorm2
 MathType@MTEF@5@5@+=feaafiart1ev1aaatCvAUfKttLearuWrP9MDH5MBPbIqV92AaeXatLxBI9gBaebbnrfifHhDYfgasaacPC6xNi=xH8viVGI8Gi=hEeeu0xXdbba9frFj0xb9qqpG0dXdb9aspeI8k8fiI+fsY=rqGqVepae9pg0db9vqaiVgFr0xfr=xfr=xc9adbaqaaeGacaGaaiaabeqaaeqabiWaaaGcbaGaemiEaG3aa0baaSqaaiabdMgaPbqaaiabd6gaUjabd+gaVjabdkhaYjabd2gaTbaakiabg2da9iabikdaYmaaCaaaleqabaGaemyqae0aaSbaaWqaaiabdMgaPbqabaWccqGHRaWkjuaGdaWcaaqaaiabd2eannaaDaaabaGaemyAaKgabaGaemOBa4Maem4Ba8MaemOCaiNaemyBa0gaaaqaaiabikdaYaaaaaaaaa@43EB@[44]. Background was estimated by the Quantarray software as the mean intensity among those pixels within the masked area between the 5^th ^and 20^th ^percentile of intensities for a given spot. Since simple background subtraction has been demonstrated to increase spot-level variability [47], no background correction was applied.

The Affymetrix GeneChip Operating System (GCOS) was used to calculate expression summaries with a target intensity of 100 using the Microarray Suite version 5.0 (MAS 5.0) method [48]. For completeness, we also estimated expression using the robust multiarray average (RMA) [49] and GC-RMA methods [50], although these methods normalize and estimate probe set expression summaries utilizing data across the entire set of GeneChips and therefore may overestimate reproducibility. All normalization and expression summary methods were performed using the R software [51] and relevant Bioconductor packages [52].

### Identifying common genes across platforms

The RESOURCERER annotation and cross-reference database [53] was developed to help investigators identify genes commonly interrogated by different microarray platforms. Other software tools such as MergeMaid [54], GeneHopper [55], MatchMiner [56], and ProbeMatchDB [57] have been developed for a similar purpose. Recent research has demonstrated improved cross-platform correlations when spots are matched by sequence rather than by gene identifiers [58-60].

Therefore, probe sets and spots with common sequences to all three platforms were retained for analysis using the following method. First, the GCG program 'netfetch' was used to obtain the NCBI GenBank records for spot IDs on the GMU and C3B microarray platforms. The perfect match (PM) probe level sequence data for the Affymetrix HG-U133A GeneChip was downloaded from the Affymetrix website (06/14/2005). BLASTN (v2.2.10) was used to query the Affymetrix probe sequences against the C3B sequences. Thereafter, all probe sets for which at least 60% of the probes reported low e-scores values (*E <*0.000001) for the same spot were retained as matches. This threshold was determined considering the breakdown bound of the Tukey biweight estimator used in the MAS 5.0 expression summary algorithm. M-estimators with symmetric ψ-function have breakdown bound close to 50%. Therefore, probe sets for which *> *60% of its PM probes specifically interrogated the same RefSeqID were retained. For the C3B microarray, each RefSeqID is spotted two times on the array. For the intra-platform reliability study (Stratagene dataset), average spot intensity per RefSeqID was retained as C3B gene expression. For the Affymetrix GeneChips, when multiple probe sets interrogated the same transcript, first, that probe set with the maximum proportion of probes with *E <*0.000001 was retained; when two or more probe sets had the same proportion, then the most 3' probe set was retained, defined by the probe set with maximum stop query sequence location among probes within a GenBank ID; when both quantities were the same, the probe set was randomly selected.

This process was completed separately for the Affy-C3B and Affy-GMU platform pairs. These two resulting datasets were merged by Affymetrix probe set ID, resulting in a dataset containing only genes in common to all three platforms.

All raw microarray files used in this study are publicly available [61].

### Intra-platform analyses

It has been suggested that poor cross-platform correlation is likely a result of low intra-platform consistency [26]. Therefore, prior to estimating cross-platform reproducibility and gene-specific reliability, intra-platform reproducibility for three different microarray platforms was examined. After normalization and calculation of gene expression summaries, within-platform correlation was estimated using average Pearson correlation for the *K *= 3 chips. In addition, reproducibility was examined by comparing the proportion of invariant genes across the set of technical replicates within a platform. Specifically, for spot *i *= 1, . . ., *G*, the ranked expression for the *k*^*th *^replicate of platform *l *is denoted by *R*_*ikl*_. We then identified the rank difference for each spot *i *within platform *l *as Δ_*il *_= *abs*(*argmax*_*il*_(*R*_*ikl*_) - *argmin*_*il*_(*R*_*ikl*_)). A gene was designated as 'invariant' for platform *l *using the indicator *I*(Δ_*il*_/*G *≤ 0.05). As an example, this would correspond to permitting the rank to shift by no more than 1,114 when 22,283 genes are spotted on the array. Statistical tests of hypothesis comparing the proportions of invariant genes across platforms were conducted using a chi-square test.

Finally, the weighted kappa statistic was estimated by first grouping gene expression intensities into 25 approximately equal-sized classes based on their ranked intensities, *y*_*i*_. A weighted kappa statistic was used to allow a smaller penalty of misclassification among closely related classes, where the weights were taken to be *w*_*rc *_= (1 - 0.1 × |*r *- *c*|) when |*r *- *c*| < 10 and 0 otherwise.

### Attenuation

When fitting a linear regression model

*y*_*i *_= *β*_0 _+ *β*_1_*x*_*i *_+ *ε*_*i*_

for observed random variables *x*_*i *_and *y*_*i *_on observations *i *= 1, ..., *n*, it is assumed *x*_*i *_~ *N*(*μ*_*x*_, σx2
 MathType@MTEF@5@5@+=feaafiart1ev1aaatCvAUfKttLearuWrP9MDH5MBPbIqV92AaeXatLxBI9gBaebbnrfifHhDYfgasaacPC6xNi=xH8viVGI8Gi=hEeeu0xXdbba9frFj0xb9qqpG0dXdb9aspeI8k8fiI+fsY=rqGqVepae9pg0db9vqaiVgFr0xfr=xfr=xc9adbaqaaeGacaGaaiaabeqaaeqabiWaaaGcbaacciGae83Wdm3aa0baaSqaaiabdIha4bqaaiabikdaYaaaaaa@3035@), *ε*_*i *_~ *N*(0, σe2
 MathType@MTEF@5@5@+=feaafiart1ev1aaatCvAUfKttLearuWrP9MDH5MBPbIqV92AaeXatLxBI9gBaebbnrfifHhDYfgasaacPC6xNi=xH8viVGI8Gi=hEeeu0xXdbba9frFj0xb9qqpG0dXdb9aspeI8k8fiI+fsY=rqGqVepae9pg0db9vqaiVgFr0xfr=xfr=xc9adbaqaaeGacaGaaiaabeqaaeqabiWaaaGcbaacciGae83Wdm3aa0baaSqaaiabdwgaLbqaaiabikdaYaaaaaa@300F@) which is independent of *x*_*i*_, and *x*_*i *_is measured without error [62]. Using the formulas for estimating Pearson's correlation and the slope parameter *β*_1_, Pearson's correlation can be shown to be

ρ^(x,y)=σ^xσ^yβ^1.
 MathType@MTEF@5@5@+=feaafiart1ev1aaatCvAUfKttLearuWrP9MDH5MBPbIqV92AaeXatLxBI9gBaebbnrfifHhDYfgasaacPC6xNi=xI8qiVKYPFjYdHaVhbbf9v8qqaqFr0xc9vqFj0dXdbba91qpepeI8k8fiI+fsY=rqGqVepae9pg0db9vqaiVgFr0xfr=xfr=xc9adbaqaaeGacaGaaiaabeqaaeqabiWaaaGcbaacciGaf8xWdiNbaKaacqGGOaakcqWG4baEcqGGSaalcqWG5bqEcqGGPaqkcqGH9aqpjuaGdaWcaaqaaiqb=n8aZzaajaWaaSbaaeaacqWG4baEaeqaaaqaaiqb=n8aZzaajaWaaSbaaeaacqWG5bqEaeqaaaaacuWFYoGygaqcamaaBaaabaGaeGymaedabeaacqGGUaGlaaa@3F95@

Therefore, Pearson's correlation measures the strength of the linear relationship between *X *and *Y*.

For a general problem, suppose *x*_*i *_cannot be measured precisely but rather is measured with error. Denote the error-prone measurements xiw
 MathType@MTEF@5@5@+=feaafiart1ev1aaatCvAUfKttLearuWrP9MDH5MBPbIqV92AaeXatLxBI9gBaebbnrfifHhDYfgasaacPC6xNi=xH8viVGI8Gi=hEeeu0xXdbba9frFj0xb9qqpG0dXdb9aspeI8k8fiI+fsY=rqGqVepae9pg0db9vqaiVgFr0xfr=xfr=xc9adbaqaaeGacaGaaiaabeqaaeqabiWaaaGcbaGaemiEaG3aaSbaaSqaaiabdMgaPnaaBaaameaacqWG3bWDaeqaaaWcbeaaaaa@3082@ = *x*_*i *_+ *u*_*i *_where *u*_*i *_~ (0, σu2
 MathType@MTEF@5@5@+=feaafiart1ev1aaatCvAUfKttLearuWrP9MDH5MBPbIqV92AaeXatLxBI9gBaebbnrfifHhDYfgasaacPC6xNi=xH8viVGI8Gi=hEeeu0xXdbba9frFj0xb9qqpG0dXdb9aspeI8k8fiI+fsY=rqGqVepae9pg0db9vqaiVgFr0xfr=xfr=xc9adbaqaaeGacaGaaiaabeqaaeqabiWaaaGcbaacciGae83Wdm3aa0baaSqaaiabdwha1bqaaiabikdaYaaaaaa@302F@). It is well known that fitting the model

yi=β0+β1∗xiw+εi
 MathType@MTEF@5@5@+=feaafiart1ev1aaatCvAUfKttLearuWrP9MDH5MBPbIqV92AaeXatLxBI9gBaebbnrfifHhDYfgasaacPC6xNi=xI8qiVKYPFjYdHaVhbbf9v8qqaqFr0xc9vqFj0dXdbba91qpepeI8k8fiI+fsY=rqGqVepae9pg0db9vqaiVgFr0xfr=xfr=xc9adbaqaaeGacaGaaiaabeqaaeqabiWaaaGcbaGaemyEaK3aaSbaaSqaaiabdMgaPbqabaGccqGH9aqpiiGacqWFYoGydaWgaaWcbaGaeGimaadabeaakiabgUcaRiab=j7aInaaBaaaleaacqaIXaqmcqGHxiIkaeqaaOGaemiEaG3aaSbaaSqaaiabdMgaPnaaBaaameaacqWG3bWDaeqaaaWcbeaakiabgUcaRiab=v7aLnaaBaaaleaacqWGPbqAaeqaaaaa@4056@

using the error-prone values xiw
 MathType@MTEF@5@5@+=feaafiart1ev1aaatCvAUfKttLearuWrP9MDH5MBPbIqV92AaeXatLxBI9gBaebbnrfifHhDYfgasaacPC6xNi=xH8viVGI8Gi=hEeeu0xXdbba9frFj0xb9qqpG0dXdb9aspeI8k8fiI+fsY=rqGqVepae9pg0db9vqaiVgFr0xfr=xfr=xc9adbaqaaeGacaGaaiaabeqaaeqabiWaaaGcbaGaemiEaG3aaSbaaSqaaiabdMgaPnaaBaaameaacqWG3bWDaeqaaaWcbeaaaaa@3082@ leads to the attenuated estimate *β*_1* _for *β*_1 _[28]. That is, the slope parameter is biased. Therefore, when fitting a simple linear regression model using the error prone measurements xiw
 MathType@MTEF@5@5@+=feaafiart1ev1aaatCvAUfKttLearuWrP9MDH5MBPbIqV92AaeXatLxBI9gBaebbnrfifHhDYfgasaacPC6xNi=xH8viVGI8Gi=hEeeu0xXdbba9frFj0xb9qqpG0dXdb9aspeI8k8fiI+fsY=rqGqVepae9pg0db9vqaiVgFr0xfr=xfr=xc9adbaqaaeGacaGaaiaabeqaaeqabiWaaaGcbaGaemiEaG3aaSbaaSqaaiabdMgaPnaaBaaameaacqWG3bWDaeqaaaWcbeaaaaa@3082@, the least-squares estimate is

*β*_1* _= *λβ*_1_,

where *β*_1 _is the true slope parameter describing the relationship between *y*_*i *_and *x*_*i *_and *λ *is the attenuation factor. The attenuation factor is given by

λ=σx2/(σx2+σu2)<1
 MathType@MTEF@5@5@+=feaafiart1ev1aaatCvAUfKttLearuWrP9MDH5MBPbIqV92AaeXatLxBI9gBaebbnrfifHhDYfgasaacPC6xNi=xI8qiVKYPFjYdHaVhbbf9v8qqaqFr0xc9vqFj0dXdbba91qpepeI8k8fiI+fsY=rqGqVepae9pg0db9vqaiVgFr0xfr=xfr=xc9adbaqaaeGacaGaaiaabeqaaeqabiWaaaGcbaacciGae83UdWMaeyypa0Jae83Wdm3aa0baaSqaaiabdIha4bqaaiabikdaYaaakiabc+caViabcIcaOiab=n8aZnaaDaaaleaacqWG4baEaeaacqaIYaGmaaGccqGHRaWkcqWFdpWCdaqhaaWcbaGaemyDauhabaGaeGOmaidaaOGaeiykaKIaeyipaWJaeGymaedaaa@416A@

and is used to estimate *β*_1 _when measurement error is present in both *X *and *Y *[28].

### Estimating cross-platform correlation

From the intra-platform results, it is clear that microarray gene expression data is subject to measurement error. When estimating cross-platform correlation, let *X *and *Y *represent the random variables for two different platforms, known to be measured with error. That is, Xiw
 MathType@MTEF@5@5@+=feaafiart1ev1aaatCvAUfKttLearuWrP9MDH5MBPbIqV92AaeXatLxBI9gBaebbnrfifHhDYfgasaacPC6xNi=xH8viVGI8Gi=hEeeu0xXdbba9frFj0xb9qqpG0dXdb9aspeI8k8fiI+fsY=rqGqVepae9pg0db9vqaiVgFr0xfr=xfr=xc9adbaqaaeGacaGaaiaabeqaaeqabiWaaaGcbaGaemiwaG1aaSbaaSqaaiabdMgaPnaaBaaameaacqWG3bWDaeqaaaWcbeaaaaa@3042@ = *X*_*i *_+ *u*_*i *_where *X*_*i *_~ *N *(*μ*_*x*_, σx2
 MathType@MTEF@5@5@+=feaafiart1ev1aaatCvAUfKttLearuWrP9MDH5MBPbIqV92AaeXatLxBI9gBaebbnrfifHhDYfgasaacPC6xNi=xH8viVGI8Gi=hEeeu0xXdbba9frFj0xb9qqpG0dXdb9aspeI8k8fiI+fsY=rqGqVepae9pg0db9vqaiVgFr0xfr=xfr=xc9adbaqaaeGacaGaaiaabeqaaeqabiWaaaGcbaacciGae83Wdm3aa0baaSqaaiabdIha4bqaaiabikdaYaaaaaa@3035@) and *u*_*i *_~ (0, σu2
 MathType@MTEF@5@5@+=feaafiart1ev1aaatCvAUfKttLearuWrP9MDH5MBPbIqV92AaeXatLxBI9gBaebbnrfifHhDYfgasaacPC6xNi=xH8viVGI8Gi=hEeeu0xXdbba9frFj0xb9qqpG0dXdb9aspeI8k8fiI+fsY=rqGqVepae9pg0db9vqaiVgFr0xfr=xfr=xc9adbaqaaeGacaGaaiaabeqaaeqabiWaaaGcbaacciGae83Wdm3aa0baaSqaaiabdwha1bqaaiabikdaYaaaaaa@302F@) while Yiw
 MathType@MTEF@5@5@+=feaafiart1ev1aaatCvAUfKttLearuWrP9MDH5MBPbIqV92AaeXatLxBI9gBaebbnrfifHhDYfgasaacPC6xNi=xH8viVGI8Gi=hEeeu0xXdbba9frFj0xb9qqpG0dXdb9aspeI8k8fiI+fsY=rqGqVepae9pg0db9vqaiVgFr0xfr=xfr=xc9adbaqaaeGacaGaaiaabeqaaeqabiWaaaGcbaGaemywaK1aaSbaaSqaaiabdMgaPnaaBaaameaacqWG3bWDaeqaaaWcbeaaaaa@3044@ = *Y*_*i *_+ *v*_*i *_where *Y*_*i *_~ *N *(*μ*_*y*_, σy2
 MathType@MTEF@5@5@+=feaafiart1ev1aaatCvAUfKttLearuWrP9MDH5MBPbIqV92AaeXatLxBI9gBaebbnrfifHhDYfgasaacPC6xNi=xH8viVGI8Gi=hEeeu0xXdbba9frFj0xb9qqpG0dXdb9aspeI8k8fiI+fsY=rqGqVepae9pg0db9vqaiVgFr0xfr=xfr=xc9adbaqaaeGacaGaaiaabeqaaeqabiWaaaGcbaacciGae83Wdm3aa0baaSqaaiabdMha5bqaaiabikdaYaaaaaa@3037@), *v*_*i *_~ (0, σv2
 MathType@MTEF@5@5@+=feaafiart1ev1aaatCvAUfKttLearuWrP9MDH5MBPbIqV92AaeXatLxBI9gBaebbnrfifHhDYfgasaacPC6xNi=xH8viVGI8Gi=hEeeu0xXdbba9frFj0xb9qqpG0dXdb9aspeI8k8fiI+fsY=rqGqVepae9pg0db9vqaiVgFr0xfr=xfr=xc9adbaqaaeGacaGaaiaabeqaaeqabiWaaaGcbaacciGae83Wdm3aa0baaSqaaiabdAha2bqaaiabikdaYaaaaaa@3031@). The average Pearson's correlation (ρ¯
 MathType@MTEF@5@5@+=feaafiart1ev1aaatCvAUfKttLearuWrP9MDH5MBPbIqV92AaeXatLxBI9gBaebbnrfifHhDYfgasaacPC6xNi=xH8viVGI8Gi=hEeeu0xXdbba9frFj0xb9qqpG0dXdb9aspeI8k8fiI+fsY=rqGqVepae9pg0db9vqaiVgFr0xfr=xfr=xc9adbaqaaeGacaGaaiaabeqaaeqabiWaaaGcbaacciGaf8xWdiNbaebaaaa@2DB2@_*w*_), which is not corrected for measurement error, can be estimated as

ρ¯w=∑j=13ρ^(x¯w,yjw)/3,
 MathType@MTEF@5@5@+=feaafiart1ev1aaatCvAUfKttLearuWrP9MDH5MBPbIqV92AaeXatLxBI9gBaebbnrfifHhDYfgasaacPC6xNi=xI8qiVKYPFjYdHaVhbbf9v8qqaqFr0xc9vqFj0dXdbba91qpepeI8k8fiI+fsY=rqGqVepae9pg0db9vqaiVgFr0xfr=xfr=xc9adbaqaaeGacaGaaiaabeqaaeqabiWaaaGcbaacciGaf8xWdiNbaebadaWgaaWcbaGaem4DaChabeaakiabg2da9maaqahabaGaf8xWdiNbaKaacqGGOaakcuWG4baEgaqeamaaBaaaleaacqWG3bWDaeqaaOGaeiilaWIaemyEaK3aaSbaaSqaaiabdQgaQnaaBaaameaacqWG3bWDaeqaaaWcbeaakiabcMcaPiabc+caViabiodaZaWcbaGaemOAaOMaeyypa0JaeGymaedabaGaeG4mamdaniabggHiLdGccqGGSaalaaa@4658@

where x¯w
 MathType@MTEF@5@5@+=feaafiart1ev1aaatCvAUfKttLearuWrP9MDH5MBPbIqV92AaeXatLxBI9gBaebbnrfifHhDYfgasaacPC6xNi=xH8viVGI8Gi=hEeeu0xXdbba9frFj0xb9qqpG0dXdb9aspeI8k8fiI+fsY=rqGqVepae9pg0db9vqaiVgFr0xfr=xfr=xc9adbaqaaeGacaGaaiaabeqaaeqabiWaaaGcbaGafmiEaGNbaebadaWgaaWcbaGaem4DaChabeaaaaa@2F07@ is the average log_2 _Affymetrix intensities and yjw
 MathType@MTEF@5@5@+=feaafiart1ev1aaatCvAUfKttLearuWrP9MDH5MBPbIqV92AaeXatLxBI9gBaebbnrfifHhDYfgasaacPC6xNi=xH8viVGI8Gi=hEeeu0xXdbba9frFj0xb9qqpG0dXdb9aspeI8k8fiI+fsY=rqGqVepae9pg0db9vqaiVgFr0xfr=xfr=xc9adbaqaaeGacaGaaiaabeqaaeqabiWaaaGcbaGaemyEaK3aaSbaaSqaaiabdQgaQnaaBaaameaacqWG3bWDaeqaaaWcbeaaaaa@3086@ is C3B or GMU expression. However, a more appropriate measure, the "disattenuated" correlation [30], can be calculated as

*ρ*_*w *_= *λ*_*p *_× *ρ*

where

λp=σxσyσx2+σu2σy2+σv2.
 MathType@MTEF@5@5@+=feaafiart1ev1aaatCvAUfKttLearuWrP9MDH5MBPbIqV92AaeXatLxBI9gBaebbnrfifHhDYfgasaacPC6xNi=xI8qiVKYPFjYdHaVhbbf9v8qqaqFr0xc9vqFj0dXdbba91qpepeI8k8fiI+fsY=rqGqVepae9pg0db9vqaiVgFr0xfr=xfr=xc9adbaqaaeGacaGaaiaabeqaaeqabiWaaaGcbaacciGae83UdW2aaSbaaSqaaiabdchaWbqabaGccqGH9aqpjuaGdaWcaaqaaiab=n8aZnaaBaaabaGaemiEaGhabeaacqWFdpWCdaWgaaqaaiabdMha5bqabaaabaWaaOaaaeaacqWFdpWCdaqhaaqaaiabdIha4bqaaiabikdaYaaacqGHRaWkcqWFdpWCdaqhaaqaaiabdwha1bqaaiabikdaYaaaaeqaamaakaaabaGae83Wdm3aa0baaeaacqWG5bqEaeaacqaIYaGmaaGaey4kaSIae83Wdm3aa0baaeaacqWG2bGDaeaacqaIYaGmaaaabeaaaaGaeiOla4caaa@4BBD@

This estimate adjusts for the bias present in estimating the correlation when measurement error is present. Estimates for *σ*_*x*_, *σ*_*u*_, *σ*_*y*_, and *σ*_*v *_were fit using the regression calibration rcal function in Stata version 9 [63]. In estimating σu2
 MathType@MTEF@5@5@+=feaafiart1ev1aaatCvAUfKttLearuWrP9MDH5MBPbIqV92AaeXatLxBI9gBaebbnrfifHhDYfgasaacPC6xNi=xH8viVGI8Gi=hEeeu0xXdbba9frFj0xb9qqpG0dXdb9aspeI8k8fiI+fsY=rqGqVepae9pg0db9vqaiVgFr0xfr=xfr=xc9adbaqaaeGacaGaaiaabeqaaeqabiWaaaGcbaacciGae83Wdm3aa0baaSqaaiabdwha1bqaaiabikdaYaaaaaa@302F@ and σv2
 MathType@MTEF@5@5@+=feaafiart1ev1aaatCvAUfKttLearuWrP9MDH5MBPbIqV92AaeXatLxBI9gBaebbnrfifHhDYfgasaacPC6xNi=xH8viVGI8Gi=hEeeu0xXdbba9frFj0xb9qqpG0dXdb9aspeI8k8fiI+fsY=rqGqVepae9pg0db9vqaiVgFr0xfr=xfr=xc9adbaqaaeGacaGaaiaabeqaaeqabiWaaaGcbaacciGae83Wdm3aa0baaSqaaiabdAha2bqaaiabikdaYaaaaaa@3031@, the repeated measurements were assumed to be unbiased for the true gene expression values. Moreover, any missing value was treated as missing at random. Previous investigators have reported high reproducibility estimates for Affymetrix expression values [14,40], therefore, we were primarily interested in estimating the correlation between Affymetrix and the custom designed arrays (C3B and GMU) that we have used in various cancer genomics projects. The disattenuated correlation, ρ¯
 MathType@MTEF@5@5@+=feaafiart1ev1aaatCvAUfKttLearuWrP9MDH5MBPbIqV92AaeXatLxBI9gBaebbnrfifHhDYfgasaacPC6xNi=xH8viVGI8Gi=hEeeu0xXdbba9frFj0xb9qqpG0dXdb9aspeI8k8fiI+fsY=rqGqVepae9pg0db9vqaiVgFr0xfr=xfr=xc9adbaqaaeGacaGaaiaabeqaaeqabiWaaaGcbaacciGaf8xWdiNbaebaaaa@2DB2@, and average Pearson correlation, ρ¯
 MathType@MTEF@5@5@+=feaafiart1ev1aaatCvAUfKttLearuWrP9MDH5MBPbIqV92AaeXatLxBI9gBaebbnrfifHhDYfgasaacPC6xNi=xH8viVGI8Gi=hEeeu0xXdbba9frFj0xb9qqpG0dXdb9aspeI8k8fiI+fsY=rqGqVepae9pg0db9vqaiVgFr0xfr=xfr=xc9adbaqaaeGacaGaaiaabeqaaeqabiWaaaGcbaacciGaf8xWdiNbaebaaaa@2DB2@_*w*_, were estimated separately for the GMU and C3B platforms relative to Affymetrix.

## Authors' contributions

KJA performed the statistical analyses and drafted the manuscript. CID, AFG, and CTG designed and performed the MDX Affymetrix quality control study. GST and TGE designed and performed the C3B quality control study. GMG designed and performed the GMU quality control study. MDC performed the BLAST search and assisted with merging the cross-platform data. All authors read and approved the final manuscript.
